# Role of DNA Methylation in Mediating Genetic Risk of Psychiatric Disorders

**DOI:** 10.3389/fpsyt.2021.596821

**Published:** 2021-04-01

**Authors:** Anna Starnawska, Ditte Demontis

**Affiliations:** ^1^Department of Biomedicine, Aarhus University, Aarhus, Denmark; ^2^The Lundbeck Foundation Initiative for Integrative Psychiatric Research, iPSYCH, Aarhus, Denmark; ^3^Center for Genomics and Personalized Medicine (CGPM), Center for Integrative Sequencing, iSEQ, Aarhus, Denmark

**Keywords:** epigenetic regulation, DNA methylation, mQTL, CpG-SNP, GWAS, psychiatric disorder

## Abstract

Psychiatric disorders are common, complex, and heritable conditions estimated to be the leading cause of disability worldwide. The last decade of research in genomics of psychiatry, performed by multinational, and multicenter collaborative efforts on hundreds of thousands of mental disorder cases and controls, provided invaluable insight into the genetic risk variants of these conditions. With increasing cohort sizes, more risk variants are predicted to be identified in the near future, but there appears to be a knowledge gap in understanding how these variants contribute to the pathophysiology of psychiatric disorders. Majority of the identified common risk single-nucleotide polymorphisms (SNPs) are non-coding but are enriched in regulatory regions of the genome. It is therefore of great interest to study the impact of identified psychiatric disorders' risk SNPs on DNA methylation, the best studied epigenetic modification, playing a pivotal role in the regulation of transcriptomic processes, brain development, and functioning. This work outlines the mechanisms through which risk SNPs can impact DNA methylation levels and provides a summary of current evidence on the role of DNA methylation in mediating the genetic risk of psychiatric disorders.

## Introduction

Psychiatric disorders, such as major depression (MD), bipolar disorder (BD), schizophrenia (SZ), attention-deficit/hyperactivity disorder (ADHD), autism spectrum disorders (ASD), and anorexia (ANX), are leading causes of disability worldwide and pose a serious challenge to healthcare systems around the world (1). All of these disorders are common and complex phenotypes known for their multifactorial molecular etiologies. Early epidemiological studies of psychiatric disorders determined them to be highly heritable conditions, with a large proportion of variation in liability attributed to additive genetic effects [~40% for MD (2), ~70% for BD (3), ~79% for SZ (4), ~74% for ADHD (5), ~83% for ASD (6), ~50–60% for ANX (7)]. Further advancements in genomics of psychiatry confirmed common and rare genetic variations to underlie these psychiatric disorders and successfully determined loci implicated in these conditions. A significant contribution to the discovery of numerous SNPs associated with the risk of psychiatric disorders was due to large genome-wide association studies (GWASs), the largest and most recent ones reporting the discovery of 102 loci for MD (8), 30 loci for BD (9), 176 loci for SZ (10), 12 loci for ADHD (11), five loci for ASD (12), and eight loci for ANX (13), passing the threshold for genome-wide significance. Once the inflection point (14) is passed, the number of genome-wide significant loci increases linearly with increasing sample size, and thus the number of GWAS-identified risk loci is expected to increase in future studies when sample sizes get larger (15). However, despite recent GWAS discoveries, the exact molecular mechanisms through which identified risk SNPs, residing in associated risk loci, contribute to psychiatric disorders remain unclear. Majority of the identified risk SNPs is located in non-coding regions of the genome and therefore are not capable of changing amino acid protein sequences. This makes it difficult to understand how their genetic variation contributes to the etiology of these complex phenotypes. The risk SNPs are however consistently reported to be significantly enriched in regulatory regions (9, 11–13, 16, 17) and, based on topological organization of the genome, linked to additional non-coding elements regulating not only proximal but also distant genes (18, 19). Along this line, a recent cross-disorder GWAS of eight common psychiatric disorders identified 109 pleiotropic loci affecting more than one psychiatric disorder (20). These risk SNPs were annotated to genes characterized by crucial regulatory role in human neurodevelopment and by their increased expression from the beginning of the second prenatal trimester (20). Altogether, these findings suggest that the identified risk SNPs, also the non-coding ones, may act through regulation of the genome to mediate the risk of psychiatric disorders. The aim of this review is to explore the putative role of DNA methylation as a regulatory mechanism through which the identified risk SNPs could contribute to the development of psychiatric disorders.

## Importance of DNA Methylation in Psychiatric Disorders

DNA methylation, the best studied epigenetic modification, is characterized by an addition of a methyl group to the fifth position of the pyrimidine ring of cytosine base, resulting in 5-methylcytosine (5mC). DNA methylation is dynamic, changes across the individual's life span, and plays a pivotal role in the regulation of human neuronal development, functioning, and survival (21, 22). From the moment of conception, DNA methylation levels in the mammalian genome are modifiable by prenatal environmental factors encountered *in utero* (such as prenatal famine, maternal folic acid supplementation, maternal stress, maternal smoking, among others) (23–28). All of these prenatal exposures have the potential to act through the epigenome to alter the expression of genes related to neuronal function and therefore influence brain development. Additionally, life events (such as childhood maltreatment, trauma, stress) (29–32) and lifestyle choices (diet, tobacco smoking, alcohol use) (25, 33–40) modify DNA methylation levels in the genome. Many of these environmental exposures are also strongly associated with psychiatric disorders' risk, giving potential for this epigenetic modification to contribute to the molecular etiology of these conditions. Interestingly, DNA methylation signatures were also associated with brain volume (41, 42), brain structure and function (22, 43, 44), as well as social (45), and cognitive functioning (46), even though the studies were performed in peripheral tissues (blood/saliva). Altogether, the scientific discoveries of impact of DNA methylation on neuronal function, brain structure, and psychiatric disorders' environmental risk factors on human methylome gave rise to numerous epigenome-wide association studies (EWASs) of mental health phenotypes. In contrast to GWAS, in EWAS, it is DNA methylation that is quantified at hundreds of thousands of loci in the human genome in order to identify differentially methylated positions and/or regions associated with a phenotype of interest (47). EWAS of psychiatric disorders identified variations in DNA methylation levels in the genome to be associated with MD, SZ, BD, ASD, ADHD, and ANX (48–59), as well as disorder trajectory and symptomatology of ADHD and MD (60, 61). Interestingly, one EWAS performed in blood samples collected already at individual's birth demonstrated differential DNA methylation in genes involved in fetal brain development and neurogenesis to be associated with psychiatric disorder diagnosis later in life among 22q11.2 deletion carriers (62). However, despite success of these studies to identify differentially methylated sites for these disorders, no clear picture has emerged until now on the role of aberrant DNA methylation levels in the pathophysiology of psychiatric disorders. This is due to difficulties in the interpretation and replication of findings from EWAS of psychiatric disorders across studies arising from relatively low sample sizes, in comparison to GWAS, and DNA methylation being tissue-, developmental stage-, and population-specific (47). Additionally, as environmental factors, stochastic events, individual's genetic background, tissue cellular heterogeneity, as well as technical effects of the methylome quantification process impact the variance of DNA methylation at measured sites, large sample sizes are required to achieve sufficient statistical power to detect true positive differentially methylated sites associated with the disorder (63, 64). Therefore, the currently ongoing large multicenter meta-analyses of EWAS results across sites, like meta-EWAS for ASD, ADHD, cognitive functioning (65–67), should provide better insight into the role of human methylome in psychiatric disorders. However, just increasing sample sizes for EWAS may not be sufficient to inform on epigenetic changes underlying psychiatric disorders. Due to the difficulty in gaining access to brain samples, and therefore low sample sizes of brain biobanks, majority of meta-EWAS analyses are still performed on methylomic data derived from peripheral tissues, most commonly from blood. As DNA methylation is tissue-specific, findings even from a large meta-EWAS may not reflect biological processes in the brain (68). This limitation calls for rigorous replication of EWAS findings from peripheral tissues in human brain samples and encourages the use of additional statistical methods in methylomic studies, e.g., Mendelian randomization, to strengthen causal inference and explore molecular mediation by DNA methylation (69).

Apart from EWASs that commonly aim to identify significant difference in DNA methylation means between cases and controls (47), an increasing number of studies explores the impact of individual's genetic background on DNA methylation levels in the genome, with special focus on risk SNPs for common disorders.

## How Can DNA Methylation be Influenced by Genetic Variation?

Individual's genetic variation can impact DNA methylation levels in the genome, making this epigenetic modification a heritable trait. Heritability of brain DNA methylation in DNA window of size 50 kb was estimated to range 3–4% and differed markedly from previously reported blood twin-based mean genome-wide heritability of 18% (70, 71). Regardless of the differences in heritability estimates, the heritable DNA methylation loci are highly enriched in open chromatin regions, DNAase I hypersensitive sites, binding sites of transcriptional repressor CTCF, and histone modifications (70, 72). This observation suggests an important epigenetic role of DNA methylation heritable loci in the regulation of chromatin accessibility and gene expression.

Currently, the best studied phenomenon through which genetic variation impacts epigenetic regulation is methylation Quantitative Trait Loci (mQTLs). The mQTL term indicates a statistically significant association between genotype at a SNP and DNA methylation level at nearby (*cis-*) or distant (*trans-*) position in the genome (73, 74), with majority of mQTLs acting in *cis-* rather than in *trans-* manner (75). mQTLs are consistently detected across different populations, developmental stages, and tissue types (76). Studies comparing intraindividual DNA methylation patterns between different brain regions and blood found that DNA methylation levels that were correlated across these tissues were likely to result from mQTLs (68, 77). Moreover, the effect of mQTL SNP genotype on DNA methylation level has often the same direction across tissues (76), and methylation measurements by array probes at sites affected by mQTLs have higher signal reliability in comparison to probes that are not (78). These findings highlight the overall stability of DNA methylation signals at loci influenced by mQTL SNPs. Therefore, studying *cis-* and *trans-*mQTL regulation of the genome by risk SNPs, in contrast to interpretation of GWAS findings only based on genomic annotation of associated SNPs to their nearest genes, may lead to the discovery of epigenetic dysregulation at nearby or distant genes and so allow for improved interpretation of their role in psychiatric disorders.

Another phenomenon by which sequence variation can impact methylation levels in the genome are CpG-SNPs, a term that relates to CpG sites that are created or destroyed by SNPs. CpG dinucleotides are highly mutable and their frequency in the human genome is already lower than expected (their occurrence is only 1% in comparison to the expected 6.25% based on the probability of 16 possible combinations of dinucleotides) (79). The main reason for this underrepresentation of CpG sites in the genome is owed to increased mutation rate of methylated cytosines due to their higher rate of spontaneous deamination in comparison to unmethylated cytosines (80). The difference in mutation rate of methylated sites does not however account for all of the mechanisms leading to this discrepancy. Deaminated methylated cytosines result in thymine, while unmethylated cytosines result in uracil, not normally occurring in the DNA, and therefore more efficiently corrected by mismatch repair mechanisms (81). Apart from disappearance of CpG sites, SNPs can also create a new CpG site and therefore provide a new locus for epigenetic regulation. CpG-SNPs are reported to play an important role in allele-specific methylation (82); however, their full impact on common diseases and their possible enrichment in regulatory elements of the genome is currently understudied due to technological constraints to investigate this phenomenon. Majority of human methylomic studies are nowadays performed with methylation arrays that require the use of bisulfite-converted DNA. Results from this array do not reliably distinguish between signals originated from bisulfite-converted unmethylated DNA (measured as T) and between mutated C to T CpG sites. Other, currently less common technologies for methylation studies, like MeDIP-seq, MBD-seq, combination of data from methylation and genotyping arrays, and candidate gene studies performed with MassArray or pyrosequencing systems, provide better identification of CpG-SNPs in the genome and their role on DNA methylation levels at the tested site. Altogether, studies using these technological approaches increasingly report CpG-SNP loci to be genomic hotspots for risk of common disorders (immune diseases, diabetes, cancer), but more research is needed to understand their molecular genome-wide role in other complex disorders (83–85).

## Evidence for the Impact of Methylation Quantitative Trait Loci on Psychiatric Disorders

Studying the impact of genetic variation on the epigenome can fill gaps in our understanding of the mechanisms by which risk SNPs, especially the non-coding ones, contribute to the risk of psychiatric disorders. mQTLs are abundant in the human brain and across various brain regions (86). Interindividual variation in brain DNA methylation levels at numerous loci in the genome was attributed to mQTL effects, and recent studies confirmed several risk SNPs for common psychiatric disorders to act as mQTLs (73). A study performed on dorsolateral prefrontal cortex samples from SZ patients and unaffected matched controls confirmed the abundance of *cis-*mQTLs in this tissue and reported the detection of mQTL interactions to be independent from the case-control status (87). As the detection of mQTLs is largely independent of the psychiatric disorder status, it indicates a common molecular mechanism conferring regulatory function of SNPs, regardless of the trait they were initially associated with. It therefore allows for an investigation of the impact of associated SNPs on epigenetic regulation in samples collected from unaffected individuals. Study of fetal brain methylomes determined mQTLs in this tissue to be enriched in GWAS risk loci of SZ and to be localized at putative causal loci (affecting the *AS3MT* gene) associated with the disorder (88). Interestingly, the estimated SZ mQTL risk loci had a larger effect on DNA methylation levels in fetal brain in comparison to their effects in adult brain tissues (prefrontal cortex, striatum, cerebellum), supporting the hypothesis of developmental origin of this disorder (88). Both differentially methylated positions associated with SZ through EWAS were enriched in SZ GWAS candidate loci, and SZ GWAS signals were reported to co-localize with blood and brain mQTLs, altogether highlighting the potential causal regulatory impact of SZ risk SNPs (49).

In the case of BD, similarly to findings from SZ, *cis-*mQTLs across the genome were found to be enriched in variants associated with BD (89). Cross-tissue *cis-*mQTLs overlapping between blood, brain, and saliva were strongly enriched in SZ-associated variants but not with BD, what might indicate higher tissue specificity in molecular pathology of BD (89). This observation was supported by an independent study where the top susceptibility variants for BD were found to be enriched in mQTLs in the cerebellum, but not in transformed lymphocytes (90). However, as the transformation alters the methylomic landscape of cells, it is unclear how this process impacts methylation levels at mQTL loci and further detection of the enrichment (91). Candidate gene study of BD risk SNPs in *CACNA1C* confirmed its risk variants to act as *cis-*mQTLs in blood (92). The study found also that variance in DNA methylation levels at *CACNA1C cis-*mQTL sites was independently associated not only with genotypes of BD risk SNPs but also with individual's sex and BD diagnosis itself (92). Therefore, to what extent DNA methylation levels at mQTL regulatory sites across the genome are modifiable by cell transformations, additional environmental exposures, and intraindividual characteristics is currently understudied, but the impact of these factors on methylome in a tissue-specific manner could hinder the detection of such cross-tissue signals in BD and other phenotypes.

In MD, representing another affective disorder, there is strong evidence that at least nine of the MD GWAS-associated risk SNPs act as *cis-*mQTLs (16). Also, a recent study that proposed a blood-based methylation risk score for MD prediction found that 71 CpG sites, out of 196 sites contributing to the predictor, were blood mQTLs during middle-age time point and 11 CpG sites regulated by mQTLs also associated with MD after false discovery rate (FDR) correction (93).

In the case of childhood-onset psychiatric disorders (ASD and ADHD), the *cis-*mQTLs in blood, brain, and fetal brain were reported among identified risk SNPs (94). Moreover, the applied mQTL approach allowed for the refinement of ADHD and ASD risk loci and identification of additional genes with altered epigenetic regulation due to risk SNPs of these disorders, not linked to these SNPs before based on annotation to the closest gene (94). ASD GWAS risk SNPs were also associated with altered methylation levels in the genome in neonatal blood, and the *cis-*mQTLs co-localized with ASD GWAS findings, highlighting potential regulatory variation to be associated with the disorder (94). Apart from the abovementioned psychiatric disorders, risk SNPs of other mental health conditions (e.g., alcohol dependence disorder) were reported to be mQTLs in post-mortem prefrontal cortex and to be enriched in GWAS-identified risk loci of the studied phenotypes (95). Additionally, a recent conditional GWAS, aimed to identify disorder-specific SNPs for five psychiatric disorders (SZ, BD, MD, ASD, ADHD), reported that the associated disorder-specific SNPs also act as *cis-*mQTLs in fetal and adult brain tissues for the conditional traits (18 methylation sites were identified for SZ, two for BD, 37 for MD, eight for ADHD, and six for ASD) (96). Overall, there is increasing evidence that the risk SNPs of psychiatric disorders impact epigenetic regulation by acting as mQTLs across tissues and developmental stages. An overview of studies providing evidence on DNA methylation changes due to mQTL SNPs or at CpG-SNP positions associated with psychiatric disorders is included in Table 1.

**Table 1:** Overview of studies providing evidence on changes in DNA methylation associated with risk SNPs for common psychiatric disorders.

**Type of association between risk SNP and DNA methylation**	**Phenotype**	**Tissue**	**Developmental stage**	**Size**	**DNA methylation quantification method**	**Study describing the association**
mQTL	Schizophrenia	Dorsolateral prefrontal cortex	Adult	*N =* 216	27K methylation array	(87)
		Fetal brain	Prenatal	*N =* 166	450K methylation array	(88)
		Blood	Adult	*N =* 639	450K methylation array	(49)
		Prefrontal cortex, blood, saliva	Adolescent, adult	*N =* 1,292	EPIC methylation array, 450K methylation array	(89)
	Bipolar disorder	Cerebellum	Adult	*N =* 153	27K methylation array	(90)
		Blood	Adult	*N =* 725	iPLEX	(92)
	Major depression	Blood	Adult	*N =* 3,284	450K methylation array	(16)
		Blood	Adult	*N =* 742	450K methylation array	(93)
	Autism	Blood	Child	*N =* 968	450K methylation array	(97)
		Blood	Neonatal, child	*N =* 1,257	450K methylation array	(65)
	Autism, attention-deficit/hyperactivity disorder, autism	Fetal brain, brain cortical tissue, blood	Prenatal, adult	*N =* 2,353	450K methylation array, EPIC methylation array	(94)
	Attention-deficit/hyperactivity disorder	Saliva	Child	*N =* 611	EPIC methylation array	(98)
	Schizophrenia, bipolar disorder, major depression, autism, attention-deficit/hyperactivity disorder	Fetal brain, brain cortical tissue	Prenatal, adult	*N =* 1,178	450K methylation array	(96)
	Alcohol dependence disorder	Prefrontal cortex	Adult	*N =* 48	450K methylation array	(95)
CpG-SNP	Major depression	Blood	Adult	*N =* 1,132	MBD-seq	(99)
	Schizophrenia	Blood, Broadman area 10	Adult	*N =* 1,474	MBD-seq	(100)
	Alcohol dependence disorder	Dorsolateral prefrontal cortex	Adult	*N =* 28	Pyrosequencing	(101)

*mQTL, methylation Quantitative Trait Locus; SNP, single-nucleotide polymorphism*.

## Evidence for the Impact Of CpG-SNPs on Psychiatric Disorders

In contrast to mQTLs, where the genotype of an SNP is associated with altered methylation level at a nearby or distant cytosine in the genome, the CpG-SNPs allow for variation in methylation levels and in sequence at the exact position of the SNP. However, due to technological constraints to reliably investigate the variation in methylation levels at CpG-SNPs, not many studies investigated these associations with psychiatric disorders and some studies investigated only the link between genotypes at CpG-SNPs and mental health phenotypes (102). An epigenetic study of CpG-SNPs that tested if groups of cases and controls with the same genotype at these sites differ in methylation levels at these positions identified 27 CpG-SNPs associated with MD and the results to be overrepresented among findings from GWAS reports of MD and MD-related phenotypes (99). A similar study utilizing the same analytical approach, but in the context of psychotic disorders, identified a CpG-SNP rs3796293 in *IL1RAP* and its altered methylation to be significantly associated with SZ and have the same direction of effect in blood and brain of SZ patients (100). A candidate gene study of CpG-SNPs in prodynorphin, associated with alcohol dependence, showed elevated methylation at these sites in the brain of human alcoholics (101). Even though there is some evidence that methylation levels at CpG-SNPs can be linked with psychiatric disorders, more studies performed on samples encompassing different phenotypes, tissues, and developmental stages are required to comprehensively evaluate the role of epigenetic regulation at these sites in molecular etiology of psychiatric disorders.

## Discussion

With the fast-growing number of identified risk SNPs associated with psychiatric disorders, there is an urgent need for an identification of the affected biological mechanisms. Especially in case of non-coding SNPs, this can be difficult to determine. It has been established that identified common risk SNPs are enriched in chromatin regulatory regions, epigenetic marks, and genes important for brain development and functioning. Recent studies provided additional evidence that the risk SNPs for psychiatric disorders can act as mQTLs, leading to an altered DNA methylation landscape in the genome. Overall, the studies show that interpretation of GWAS results can benefit from including mQTL approaches in fine-mapping of risk loci to identify causal variants and variant prioritization. However, in order to fully understand the impact of risk SNP mQTLs on the development and trajectory of psychiatric disorders, more systematic studies on the human methylome are needed. For example, the abovementioned studies of risk SNPs and mQTLs in psychiatric disorders utilized data generated from different versions of methylation arrays (differing by types of genomic regulatory regions that they cover and number of loci tested), tissues (fetal brain, adult brain, different brain regions, blood, saliva), and developmental stages (fetal development, neonatal, adult, middle-aged). These mQTLs were also calculated based on cohorts with varying sample sizes, which may limit statistical power to detect some mQTLs, and therefore reported mQTLs may not be directly comparable across the studies. The mQTL datasets used in each study were specifically chosen to test particular hypotheses relevant for each investigated disorder, but there is still a lack of comprehensive studies of psychiatric disorder risk SNP mQTLs across lifetime and tissues. Such studies should be performed with the largest methylation arrays or whole-genome methylomic data, if possible, to provide more information on putative mQTL-regulated loci. It is also worth noting that the majority of studies investigated only *cis-*mQTL regulation, which is more abundant in the human genome than *trans-*, but lacks information on long distance or across-chromosome impact of risk SNPs on variation in methylation.

**Figure 1:**
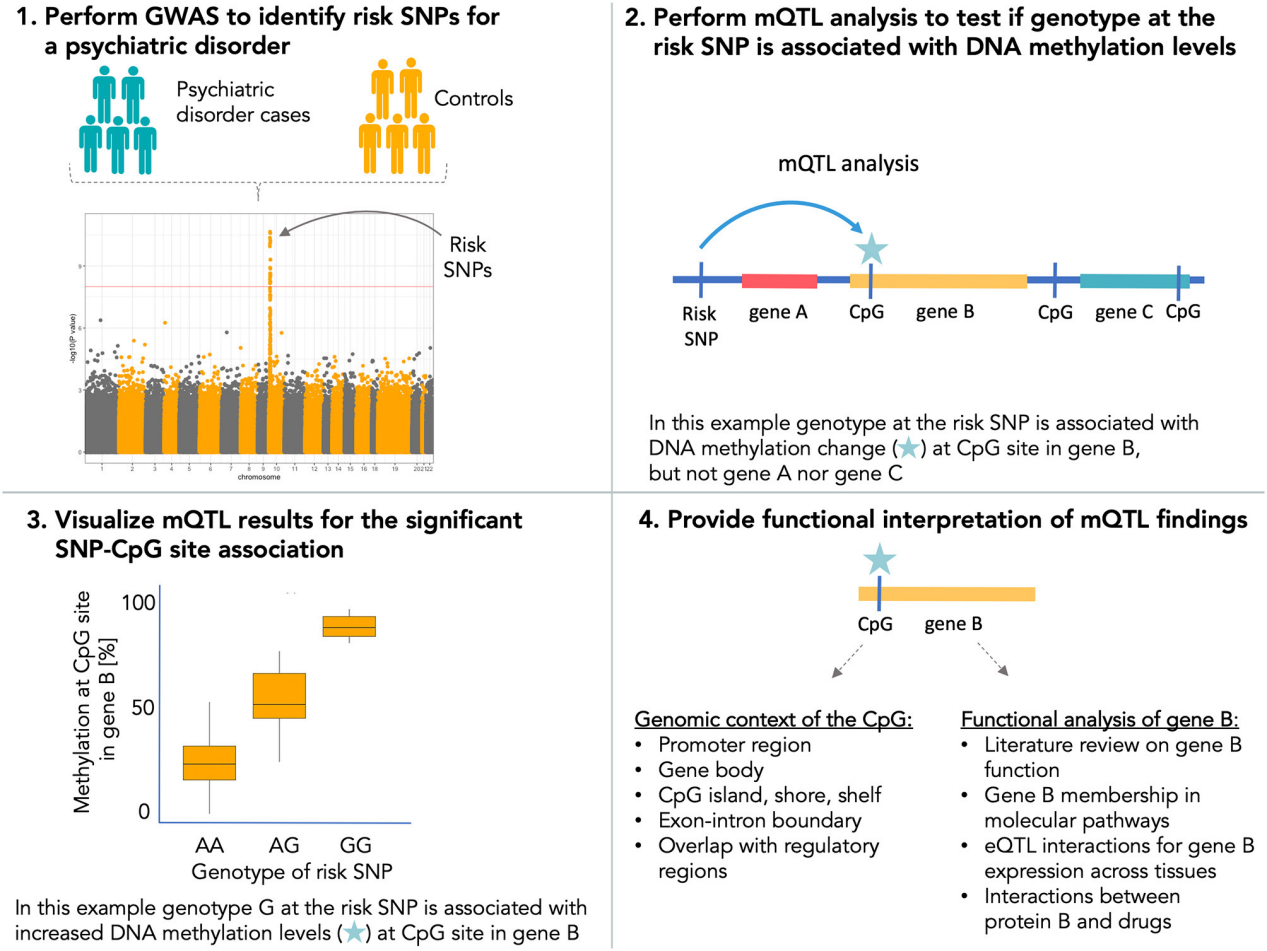
Recommended workflow for using DNA methylation data for interpretation of the functional impact of risk single-nucleotide polymorphisms (SNPs) for psychiatric disorders.

Apart from the function of risk SNPs for psychiatric disorders as mQTLs, it is also important to study expression QTLs (eQTLs) to gain insight into downstream effects of variation in DNA methylation due to risk SNPs on gene expression. DNA methylation plays a pivotal role in the regulation of transcriptional processes such as gene expression, alternative promoter usage, as well as gene splicing. It is established that mQTLs co-localize with eQTLs (86, 103–105) and fetal brain mQTLs show significant overlap with SNPs also associated with gene expression in the brain (88). A recent study performed in blood and fetal and adult brain provided also evidence for pleiotropic effects of associated mental disorder common risk SNPs (for ADHD and ASD) on DNA methylation levels and gene expression (94). All this emerging evidence points to DNA methylation as a putative regulatory mechanism to mediate mental disorder risk from associated SNPs. We therefore recommend that in order to gain deeper understanding of the functional role of risk SNPs in psychiatric disorders, GWAS findings should be subjected to mQTL analysis (Figure 1). Results from mQTL analysis should be further interpreted to study (i) the genomic context of identified CpG sites to evaluate their putative role on gene expression, splicing patterns, and impact on other regulatory elements of the genome and (ii) the molecular function of genes annotated to CpG sites with altered methylation patterns (Figure 1). Findings from mQTL analysis should also be coupled with eQTL information by, e.g., co-localization analysis to provide a more complete overview of the functional impact of risk SNPs.

These recommendations overall highlight the importance of combining genomic, environmental, expression, and epigenomic data to study psychiatric phenotypes. The scientific community therefore should work toward performing such multi-omics studies in large sample sizes across multiple relevant tissues, developmental stages, and populations to gain a deeper understanding of the complex multifactorial interplay influencing the development and trajectories of mental health phenotypes.

In conclusion, studies on how risk SNPs affect intermediate phenotypes, like DNA methylation or gene expression through mQTLs and eQTLs and how these in turn vary with complex human diseases provide valuable insight into disease etiology and may provide new knowledge for the development of future treatment or preventive approaches for psychiatric disorders.

## Author Contributions

AS wrote the first draft. DD contributed with invaluable discussions, information, and manuscript review. Both authors contributed to writing of the final version of the manuscript and approved the submitted version.

## Conflict of Interest

The authors declare that the research was conducted in the absence of any commercial or financial relationships that could be construed as a potential conflict of interest.
